# *POLG1 *p.R722H mutation associated with multiple mtDNA deletions and a neurological phenotype

**DOI:** 10.1186/1471-2377-10-29

**Published:** 2010-05-03

**Authors:** Tuomas Komulainen, Reetta Hinttala, Mikko Kärppä, Leila Pajunen, Saara Finnilä, Hannu Tuominen, Heikki Rantala, Ilmo Hassinen, Kari Majamaa, Johanna Uusimaa

**Affiliations:** 1 Department of Pediatrics, University of Oulu, Box 5000, FIN-90014, University of Oulu, Oulu, Finland; 2 Department of Neurology, University of Oulu, Box 5000, FIN-90014, University of Oulu, Oulu, Finland; 3 Department of Clinical Genetics, Oulu University Hospital, Box 5000, FIN-90014, University of Oulu, Oulu, Finland; 4 Department of Pathology, University of Oulu, Box 5000, FIN-90014, University of Oulu, Oulu, Finland; 5 Department of Medical Biochemistry and Molecular Biology, University of Oulu, Box 5000 (Aapistie 7), FIN-90014, University of Oulu, Oulu, Finland; 6 Clinical Research Center, Oulu University Hospital, Box 5000, FIN-90014, University of Oulu, Oulu, Finland

## Abstract

**Background:**

The c.2447G>A (p.R722H) mutation in the gene *POLG1 *of the catalytic subunit of human mitochondrial polymerase gamma has been previously found in a few occasions but its pathogenicity has remained uncertain. We set out to ascertain its contribution to neuromuscular disease.

**Methods:**

Probands from two families with probable mitochondrial disease were examined clinically, muscle and buccal epithelial DNA were analyzed for mtDNA deletions, and the *POLG1, POLG2, ANT1 *and *Twinkle *genes were sequenced.

**Results:**

An adult proband presented with progressive external ophthalmoplegia, sensorineural hearing impairment, diabetes mellitus, dysphagia, a limb myopathy and dementia. Brain MRI showed central and cortical atrophy, and ^18^F-deoxyglucose PET revealed reduced glucose uptake. Histochemical analysis of muscle disclosed ragged red fibers and cytochrome c oxidase-negative fibers. Electron microscopy showed subsarcolemmal aggregates of morphologically normal mitochondria. Multiple mtDNA deletions were found in the muscle, and sequencing of the *POLG1 *gene revealed a homozygous c.2447G>A (p.R722H) mutation. His two siblings were also homozygous with respect to the p.R722H mutation and presented with dementia and sensorineural hearing impairment. In another family the p.R722H mutation was found as compound heterozygosity with the common p.W748S mutation in two siblings with mental retardation, ptosis, epilepsy and psychiatric symptoms. The estimated carrier frequency of the p.R722H mutation was 1:135 in the Finnish population. No mutations in *POLG2*, *ANT1 *and *Twinkle *genes were found. Analysis of the POLG1 sequence by homology modeling supported the notion that the p.R722H mutation is pathogenic.

**Conclusions:**

The recessive c.2447G>A (p.R722H) mutation in the linker region of the *POLG1 *gene is pathogenic for multiple mtDNA deletions in muscle and is associated with a late-onset neurological phenotype as a homozygous state. The onset of the disease can be earlier in compound heterozygotes.

## Background

DNA polymerase γ (pol γ) is the only polymerase responsible for the synthesis and repair of mitochondrial DNA in mammalian cells [1,2]. The human mitochondrial DNA polymerase is a 195 kDa heterotrimer consisting of a 140 kDa catalytic subunit (pol γA) and two identical 55 kDa accessory subunits (pol γB) [3]. The C-terminus of the catalytic subunit PolγA is the *pol *domain, which is responsible for the polymerase function, while the N-terminus is responsible for exonuclease activity and proofreading of the mitochondrial DNA (mtDNA). The linker mediates a focal contact with the dimeric accessory subunit [4]. PolγA is encoded by the *POLG1 *gene [RefSeq:NM_002693].

Numerous mutations in the *POLG1 *have been described recently, with various clinical presentations [5,6] including autosomal dominant and autosomal recessive familial external ophthalmoplegia (PEO) [7-10], autosomal recessive sensory ataxic neuropathy with dysarthria and ophthalmoplegia (SANDO) [11], a mixed sensory and cerebellar ataxic syndrome with epilepsy [12,13], parkinsonism [14,15] and Alpers' hepatocerebral syndrome [16-19]. Other conditions associated with *POLG1 *mutations include male subfertility, premature menopause and cataracts [15,20]. *POLG1 *mutations can cause deletions or depletion in mtDNA [16,21-23].

We studied here the molecular etiology of a clinically probable mitochondrial disease in five patients from two Finnish families. In one family (Family A), an adult patient presented with progressive external ophthalmoplegia, sensorineural hearing impairment, diabetes mellitus and dementia. In the other family (Family B), a child presented with mental retardation, ptosis, epilepsy and psychiatric problems.

## Methods

### Patients

Patient A1 is an 83-year-old man with sensorineural hearing impairment, type 2 diabetes mellitus, dysphagia and external ophthalmoplegia. A hearing aid had been provided at age 72 years. At age 77 years, MMSE score was 19 and a neuropsychological examination revealed a mixed-type dementia. Neurological examination at age 83 years revealed external ophthalmoplegia and bilateral ptosis. Tendon reflexes in the lower limbs were absent and muscles were scanty, but muscle strength was normal. The MMSE score was 8. His ambulatory capacity was normal, but he needed help in most of his daily activities due to dementia.

**Figure 1:**
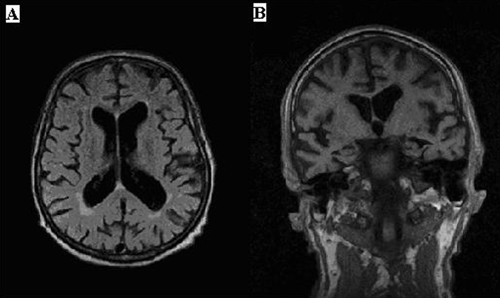
**The brain MRI of patient A1**. Axial (A) and coronal (B) MRI images of the brain of patient A1 demonstrate moderate central and cortical atrophy.

Laboratory investigations including blood lactate, pyruvate and creatine kinase were normal, as were the EEG recording and electrophysiological examination of the peripheral nerves. Brain MRI demonstrated moderate cortical and central atrophy (Figure 1), including hippocampal atrophy. Periventricular hyperintensities and a left basal ganglia infarct were also found. PET performed with ^18^F-deoxyglucose (FDG-PET) showed a reduction in glucose uptake in the frontal, frontotemporal and frontoparietal regions (data not shown). Atrophic angular, mostly type 2 muscle fibers were seen in a histological examination of the left quadriceps muscle. Histochemical analysis disclosed ragged red fibers and cytochrome oxidase-negative fibers. Electron microscopy showed subsarcolemmal aggregates of morphologically normal mitochondria (Table 1).

**Table 1:** Summary of the histological and histochemical findings of the muscle biopsy of patient A1.

**Histological findings**	Light microsopy: Atrophic, angular muscle fibers
	Electron microscopy: subsarcolemmal aggregates, morphologically normal mitochondria
**Histochemical findings**	Ragged red fibers
	Cytochrome c oxidase negative fibers

Patient A2 is the younger sister of patient A1. She is a 78-year-old woman with a history of hypertension, sensorineural hearing impairment requiring a hearing aid, hypercholesterolemia, bilateral cataract and chronic gastritis. She had been complaining of occasional headaches since the age of 30 years. A neurological examination had been performed at age 72 years on account of memory impairment. There were no focal findings in the neurological examination. Brain CT revealed cortical atrophy and one frontal lacunar infarct. The MMSE score was 26 and the score declined to 20 during the three subsequent years. Her dementia, which involved behavioural changes including aggressive symptoms and paranoid delusions, was regarded as being of an Alzheimer-like type, and cholinergic therapy was introduced. Blood lactate was slightly elevated (2.6 mmol/l, normal range 0.33-1.33 mmol/l), but blood pyruvate was normal (71 μmol/l, normal range 30-80 μmol/l), so that the lactate-pyruvate ratio was increased (37, normal < 20). The remaining laboratory tests were normal.

Patient A3 is the elder sister of patient A1. She is an 86 year-old woman with a clinical history of hypertension, type 2 diabetes mellitus, sensorineural hearing impairment, osteoarthritis, hypercholesterolemia and coronary heart disease. She had a mild left motor hemiparesis at the age of 74 years due to a lacunar brain infarct. Memory impairment appeared and mild dementia was diagnosed by a general practitioner, with CERAD and an MMSE score of 22. There were no focal neurological signs except for an absence of tendon reflexes. Brain CT revealed lacunar changes in the right hemisphere but no atrophic findings.

Patient B1 is the first child of healthy non-consanguineous parents. She was born after an uncomplicated full-term pregnancy and normal delivery. The perinatal period was uneventful and control examinations in early childhood did not reveal any abnormalities in psychomotor development, growth or hearing except for slight visual impairment. Learning difficulties were noticed after attending school for some time and she was transferred to a special class for children with learning disabilities. Seizures occurred at age 11 years, and focal generalized epilepsy was diagnosed. Her EEG was abnormal, showing focal bilateral frontotemporal irritation with secondary generalization, but a brain CT was normal. No local neurological defects were found in the follow-up clinical examination except for mild bilateral ptosis. Pubertal development was normal and she has normal menstruations. However, she gradually developed behavioral and psychiatric problems, including depression, aggressivity and suicidality and had some psychotic episodes needing hospitalization. She has no somatic health problems except for occasional cardiac arrhythmia and mild obesity. At present she is a 22-year-old woman learning to live independently, but still needs support in her daily life due to mild mental retardation and some behavioral problems.

Patient B2 is the younger sister of patient B1. She was born after a normal full-term pregnancy and delivery. Early psychomotor development was normal, but mild mental retardation was diagnosed before school age. She attended a special school for children with learning disabilities. Her pubertal development was normal, but she is mildly obese and has a mild bilateral ptosis. Like her elder sister, she has psychiatric problems, including depression, aggressivity and suicidality. At present she is a 17-year-old young woman living in a residential school for people with learning disabilities.

### Clinical evaluation

**Figure 2:**
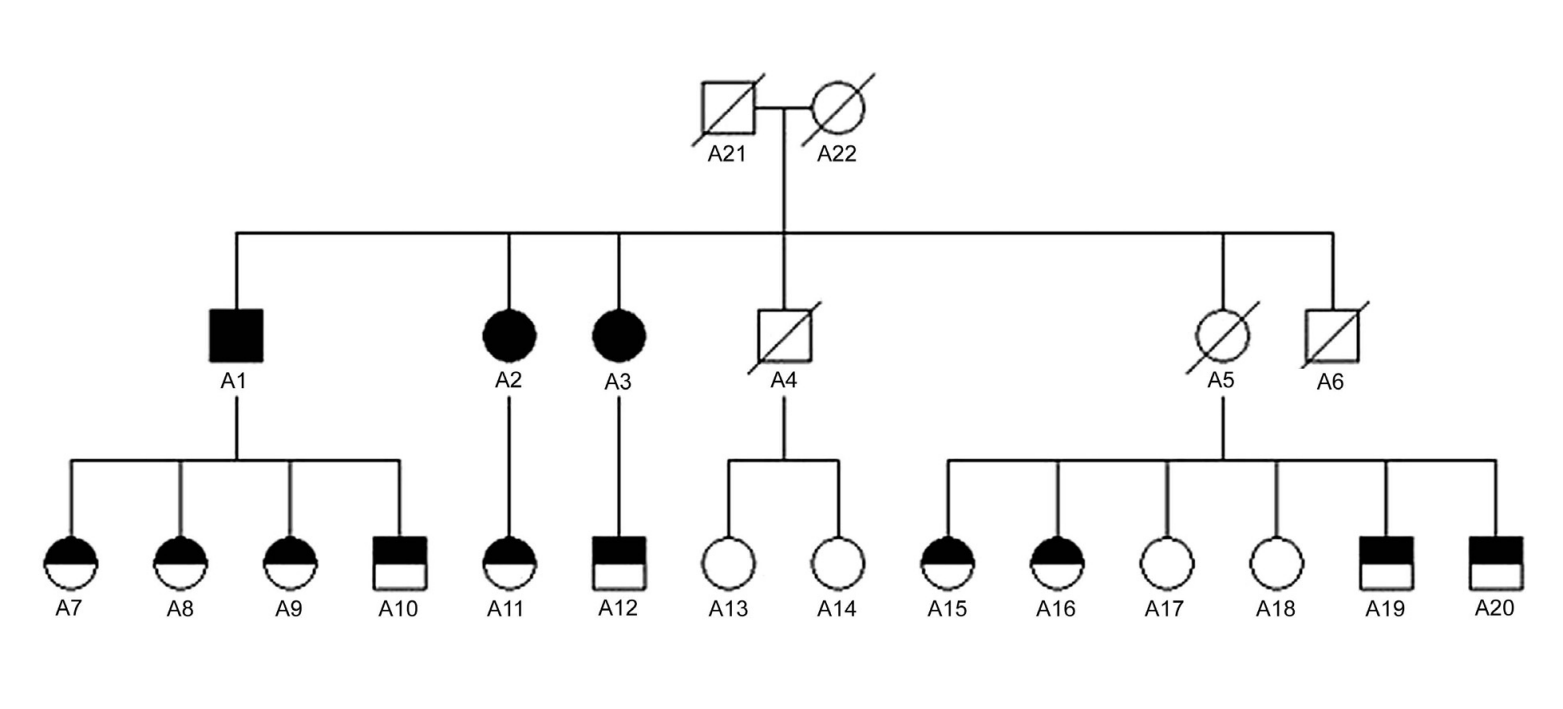
**Pedigree of family A**. Fully shaded squares (men) and circles (women) denote homozygous c.2447G>A (p.R722H) mutations in the *POLG1 *gene and half-shaded symbols heterozygosity.

Patients A1, A3, B1 and B2 were evaluated clinically by means of a neurologic examination, laboratory tests and long PCR of buccal DNA for possible mtDNA deletions. The medical charts of patient A2 were reviewed. Fourteen members of family A (Figure 2) were interviewed using a questionnaire.

### Molecular methods

Total genomic DNA was extracted from blood, muscle and buccal smear samples using the standard sodium dodecyl sulphate-proteinase K method or a QIAamp Blood Kit (Qiagen, Hilden, Germany) according to the manufacturer's instructions.

### Detection of mtDNA deletions

MtDNA deletions were analyzed by long PCR (XL-PCR) carried out using the Expand Long Template PCR System kit (Boehringer Mannheim, Mannheim, Germany) as described earlier [24].

### Analysis of the *POLG1*, *POLG2*, *ANT1 *and *Twinkle *genes

Blood DNA was used as a template to amplify and sequence the 23 coding exons of the *POLG1 *gene [RefSeq:NM_002693] in the probands (patients A1 and B1) by automated sequencing (ABI PRISM™ 377 Sequencer) using the Dye Terminator Cycle Sequencing Ready Kit (Perkin Elmer, Foster City, CA, U.S.A.) after treatment with exonuclease I and shrimp alkaline phosphatase. The entire coding sequence of the *POLG2, ANT1 *and *Twinkle *genes were also determined.

Southern blot analysis was carried out using a biotin-labeled probe. Total genomic DNA extract was digested with FastDigest PvuII (Fermentas, Burlington, ON, Canada). The mtDNA probe was amplified with PCR between nucleotide positions 15714 and 725 to cover the D-loop area, which is rarely involved in mtDNA deletions. PCR reaction included biotin-16-dUTP (Roche, Mannheim, Germany). The nylon membrane filter (Millipore, Billerica, ME, U.S.A.) was prehybridized, hybridized with the probe, washed and incubated with Odyssey Blocking Buffer (LI-COR Biosciences, Lincoln, NE, U.S.A.) and Streptavidin-IRDye 800CW conjugate (LI-COR Biosciences). The blot was scanned with Odyssey Infrared Imaging System (LI-COR Biosciences).

### Sequence analysis and molecular modeling

Secondary structure of pol γA was analyzed with the Jpred3 server [25]. Sequence alignment of DNA polymerase γA of *Homo sapiens *(human), *Pan troglodytes *(chimpanzee), *Canis lupus familiaris *(dog), *Mus musculus *(house mouse), *Rattus norvegicus *(rat), *Gallus gallus *(chicken), *Danio rerio *(zebrafish) and *Drosophila melanogaster *(fruit fly) was carried out with the ClustalW program.

Molecular modeling of the wild-type or R722H mutation-carrying DNA polymerase-γA protein was performed with the Modeller (v. 8.2) [26] program by using the 3-D structure of the Klenow fragment of *Escherichia coli *DNA polymerase I (1kln.pdb) as a template, and the results were visualized with the MOLMOL program [27].

### Detection of the *POLG1 *c.2447G>A (p.R722H) mutation

The *POLG1 *c.2447G>A (p.R722H) mutation was detected by restriction fragment analysis using a mismatch primer that creates a restriction site for MlsI (BalII) in the presence of the mutation. The mismatch primer was designed in a forward direction with the sequence 5'-TGTGTGGCCCTCACAGACTGGCC-3' (mismatch base underlined). The sequence of the reverse primer was 5'-TCAGGTGTGTCACTCTGAAGG-3'. Template DNA was amplified by PCR, digested and the products electrophoresed on a 3% MetaPhor (Cambrex Bio Science Rockland, Inc., Rockland, ME, U.S.A.) gel stained with ethidium bromide. The wild-type allele yields a 221-bp band and the mutant allele a 183-bp band.

### Detection of the POLG1 c.2243G>C (p.W748S) mutation

Allele-specific amplification was used to verify the c.2243G>C (p.W748S) mutation in the blood DNA of patients B1 and B2 and their mother. The primers, containing a locked nucleic acid (LNA) nucleoside base at the 3' end (Proligo LLC, Paris, France), were designed to anneal with either the wild-type sequence or the sequence containing the mutation [28]. The sequence of the LNA Polg14F/G primer was 5'-GGACATCCCTGGCTGCT+G-3' and of the LNA Polg14F/C 5'-GGACATCCCTGGCTGCT+C-3'. The sequence of the reverse primer was 5'-TCAGGTGTGTCACTCTGAAGG-3'.

### Ethical considerations

The study protocol has been approved by Ethical Committee of Faculty of Medicine, University of Oulu and is in compliance with the Helsinki Declaration. Written informed consent is given by all patients or their surrogates prior to the study.

## Results

### Clinical findings

Clinical examination of patient A1 and patient A3 and the review of the medical files of patient A2 revealed dementia, sensorineural hearing impairment, progressive external ophthalmoplegia and ptosis. The blood lactate/pyruvate ratio was elevated in patient A2, but not in patients A1 and A3, and the cerebral glucose metabolism of patient A1 was reduced to 20-30%. The brain MRI of patient A1 (Figure 1) showed moderate central and cortical atrophy, while that of patient A2 revealed cortical atrophy and one frontal lacunar infarct. A summary of the medical histories of ten *POLG1 p.R722H *mutation carriers, five deceased individuals and four non-carriers in the family A is presented in Table 2.

**Table 2:** Summary of the medical histories of members of family A with p.R722H mutation in *POLG1*.

Family member code	Clinical manifestation
**p.R722H homozygotes**	

Patient A1	Severe dementia, sensorineural hearing impairment, diabetes mellitus, PEO, dysphagia, neuropathic pain in the legs
Patient A2	Moderate dementia, sensorineural hearing impairment, occasional headaches, cataract
Patient A3	Mild dementia, sensorineural hearing impairment, diabetes mellitus, hypertension, coronary heart disease, areflexia due to diabetic neuropathy

**Carriers of p.R722H**	

A7	Diabetes mellitus, hypothyreosis, cerebral infarction, hypertension, hypercholesterolemia, learning difficulties
A8	Transient hypertension, benign cardiac arrhythmias, fertility problems
A9	Hypothyreosis, gestational diabetes mellitus
A10	Mental retardation
A11	Premature puberty, short stature, fertility problems, gestational diabetes mellitus
A12	Tinnitus, benign cardiac arrhythmias
A15	Cataract, balance disturbances, retinal rupture, tinnitus, unilateral deafness
A16	Coronary heart disease, delayed puberty, sensorineural hearing loss
A19	Delayed puberty, hypogonadism, transient vertigo, visual field defect
A20	Healthy

**Deceased individuals of unknown mutation status**	

A4	Dementia, coronary heart disease, diabetes mellitus, rheumatoid arthritis, operated for colon cancer
A5	Diabetes mellitus, bradycardia, sensorineural hearing loss, coronary heart disease
A6	Psychiatric problems, sudden death from intracerebral haemorrhage
A21	Impaired memory, sudden death from cerebral infarct
A22	Dementia, hemiplegia progressing to diplegia, diabetes mellitus

**Non-carriers**	

A13	Hypertension, distal sensory impairment in the hand
A14	Hypertension
A17	Unilateral hearing loss due to chronic secretory otitis, benign cardiac arrhythmia
A18	Healthy

Patient B1 presented with mental retardation, psychiatric symptoms, mild bilateral ptosis and epilepsy, while her sister (patient B2) had mental retardation, psychiatric symptoms and mild bilateral ptosis, but no epilepsy. Their mother had a history of mitral valve insufficiency, which was operated on about three years ago, chronic atrial fibrillation, for which she receives permanent anticoagulant therapy, cognitive impairment and impaired hearing, but no localizing neurological symptoms. Her MMSE score was 27. The blood lactate/pyruvate ratios of patients B1 and B2 and their mother were normal. Medical history of the father was not available.

### Molecular genetics

**Figure 3:**
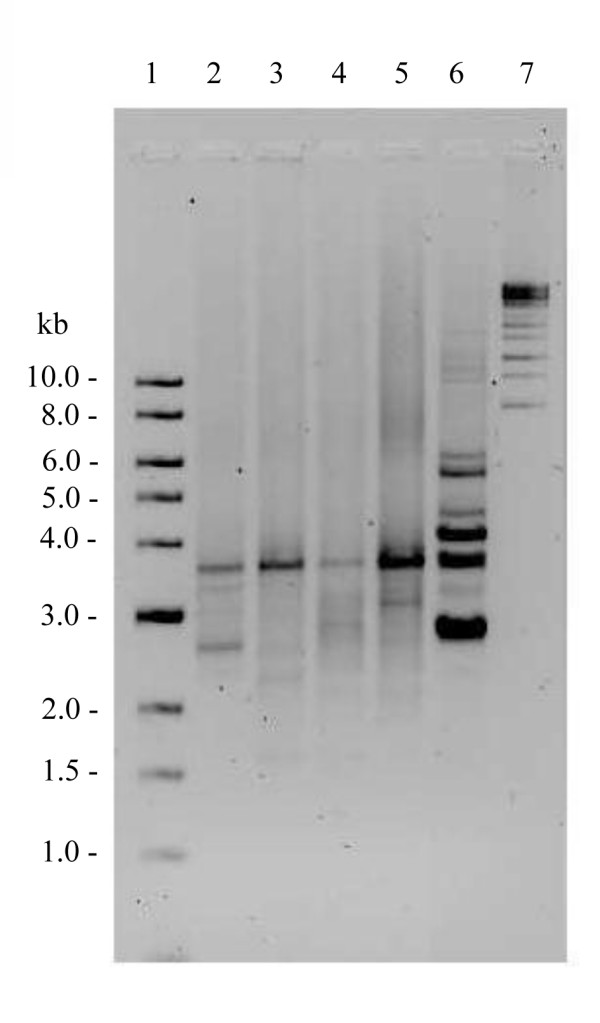
**Long PCR of muscle DNA from patient A1 with *POLG1 *p.R722H mutation**. Lane 1, 1 kb ladder; lane 2, control muscle DNA, age 80 years; lane 3, control muscle DNA, age 89 years; lane 4, control muscle DNA, age 86 years; lane 5, control muscle DNA, age 75 years; lane 6, muscle DNA from patient A1, showing multiple mtDNA deletions; lane 7, λ mix ladder.

**Figure 4:**
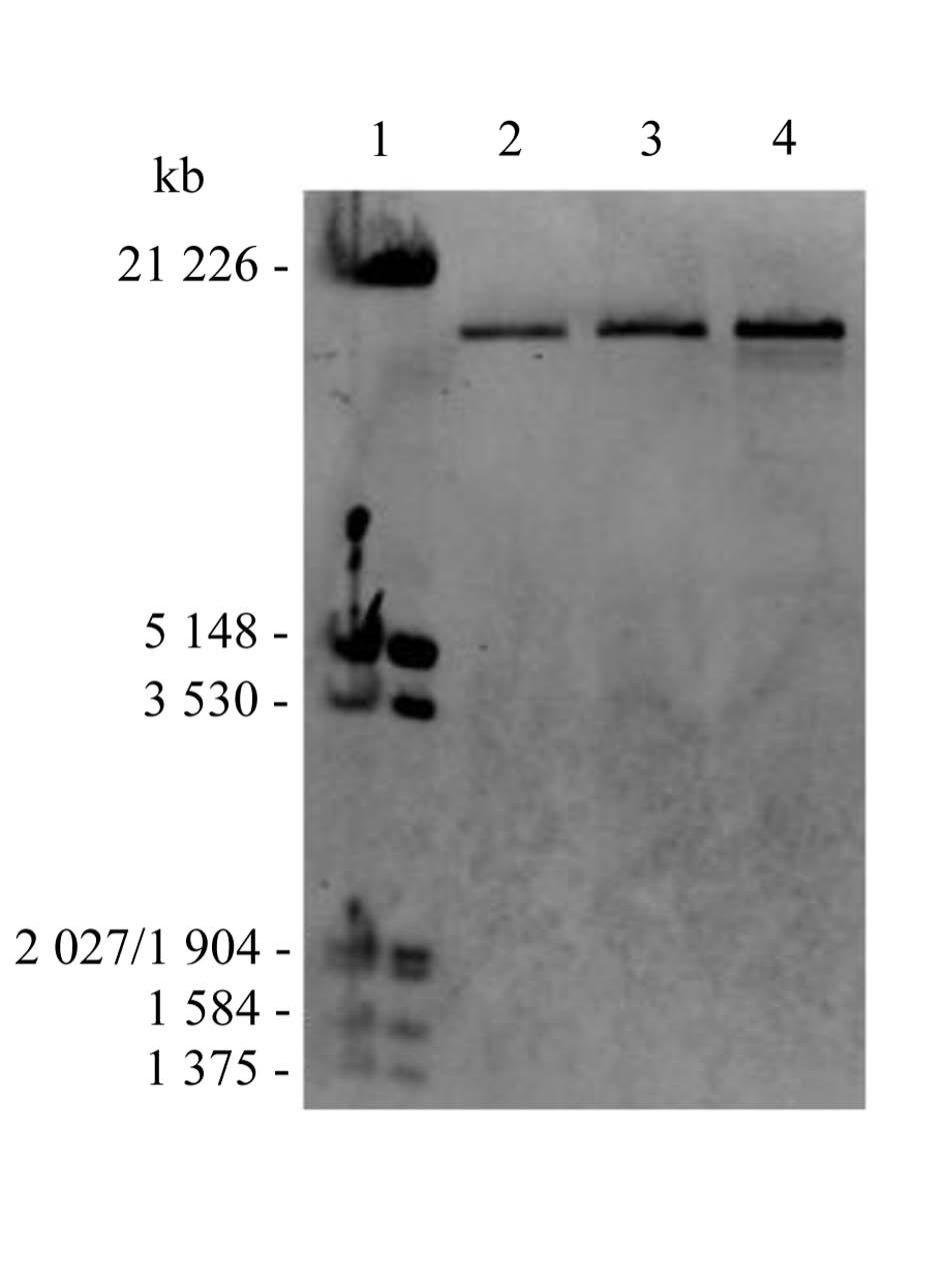
**Southern blot analysis of muscle DNA from patient A1**. Lane 1, Biotin-labeled Lambda DNA/*Eco*R1 + *Hind*III ladder; lane 2, control muscle DNA, age 80 years; lane 3, control muscle DNA, age 89 years; lane 4, patient A1.

**Figure 5:**
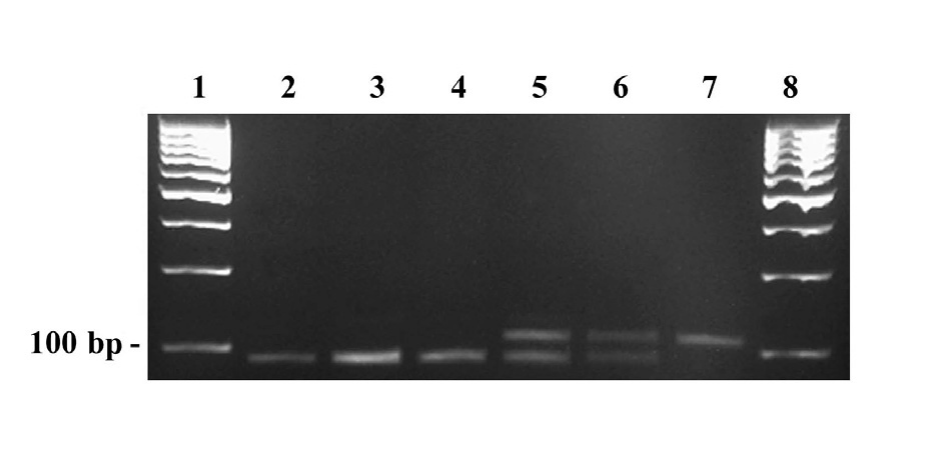
**Detection of the c.2447G>A (p.R722H) mutation in the *POLG1 *gene**. *POLG1 *p.R722H mutation was detected from blood samples by restriction fragment analysis using the MlsI (BalII) enzyme. The 183 bp fragment is visible in the case of the c.2447G>A (p.R722H) allele and the 221 bp fragment in the case of the wild-type allele. Lane 1, 100 bp ladder; lane 2, Patient A1; lane 3, Patient A2; lane 4, Patient A3; lane 5, Patient B1; lane 6, Patient B2; lane 7, Mother of patients B1 and B2.

Long PCR of the muscle DNA of patient A1 showed multiple mtDNA deletions (Figure 3), and subsequent sequencing of the entire *POLG1 *gene revealed a homozygous c.2447G>A (p.R722H) transition in exon 13. Long PCR of the muscle of age-matched controls showed deletions in minor extent, but no deletions were detected with Southern blot analysis (Figure 4). The homozygous *POLG1 *p.R722H mutation of patient A1 was also found in patients A2 and A3 (Figure 5), but no mtDNA deletions were detected in the buccal or blood DNA of these patients (data not shown). Muscle DNA was not available for analysis from patients A2 and A3. No mutations were found in the *POLG2, ANT1 *and *Twinkle *genes analyzed from blood DNA of patients A1 and A2.

A heterozygous p.R722H mutation was found in 10 out of 17 members of family A, and four members were homozygous with respect to the wild type (Figure 2). There were also five deceased individuals in this family that could be evaluated. Subject A5 had been an obligatory carrier and A21 and A22 had been carriers, or else one of them could have had a homozygous mutation, and therefore subject A4 could have been either a carrier or non-carrier of the mutation.

**Figure 6:**
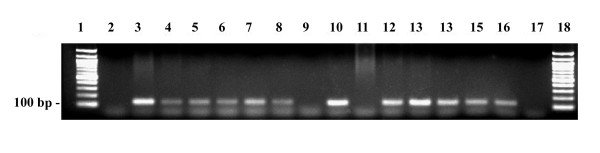
**Detection of the c.2243G>C (p.W748S) mutation in the *POLG1 *gene**. *POLG1 *p.W748S mutation was detected from blood and buccal smear samples by LNA-primers. Lanes 2-9 were obtained with the Polg14F/C primer, showing one 140 bp band in the case of the mutation, and lanes 10-17 with the Polg14F/G primer, showing a 140 bp band in the case of the wild-type. Lane 1, 100 bp ladder; lane 2, wild-type control; lane 3, positive mutation control; lane 4, heterozygous p.W748S control sample; lane 5, blood sample from patient B1; lane 6, buccal smear sample from B1; lane 7, mother; lane 8, patient B2; lane 9, H_2_O; lane 10, wild-type control; lane 11, positive mutation control; lane 12, heterozygous p.W748S control sample; lane 13, blood sample from patient B1; lane 14, buccal smear sample from patient B1; lane 15, mother; lane 16, patient B2; lane 17, H_2_O; lane 18, 100 bp ladder.

Patient B1 and patient B2 were heterozygous with respect to c.2447G>A (p.R722H) and, interestingly, *POLG1 *sequencing also revealed the c.2243G>C (p.W748S) mutation and p.E1143G polymorphism in patient B1 (Figure 5 and 6). *POLG1 *of patient B2 was not sequenced. Their mother harbored heterozygous p.W748S (Figure 5 and 6), but no DNA was available for analysis from the father or other relatives. Long PCR of buccal DNA did not show any deletions in the mtDNA of patient B1. Three samples from 403 anonymous blood donors harbored the heterozygous c.2447G>A (p.R722H), suggesting a carrier frequency of 1:135 in the population.

### Sequence analysis and molecular modeling

**Figure 7:**
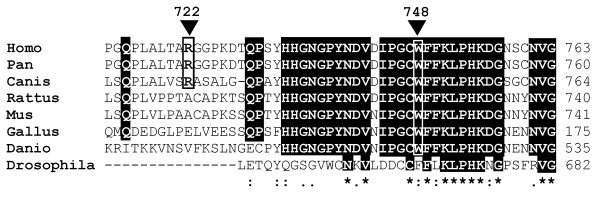
**Sequence alignment of part of the spacer regions of DNA polymerase γA**. The mutation sites p.R722H and p.W748S are marked with arrowheads. The sequences (and accession numbers) are: human [RefSeq:NP_001119603.1], Pan troglodytes [RefSeq:XP_523149.2], Canis familaris [RefSeq:XP_545850.2], Rattus Norwegicus [RefSeq:NP_445980.1], Mus musculus [GenBank:AAA98977.1], Gallus gallus [GenBank:AAC60018.1], Danio rerio [RefSeq:XP_001921130.1] Drosophila melanogaster [GenBank:AAF53338.1].

**Figure 8:**
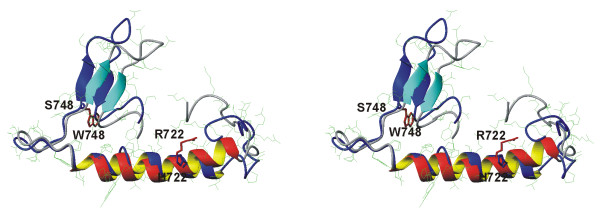
**Cartoon of a tentative structure of a segment of the spacer region of human pol γA**. Stereo views of the region from residue #700 to #770 of the normal (red/gray) and p.R722H/p.W748S double mutant (blue) protein is shown. The structure was predicted with homology modeling by using 1kln.pdb as a template. p.R722 (red), p.W748 (red), p.H722 (blue) and p.S748 (blue) are highlighted.

Although the p.R722H and p.W748S mutations analyzed in this study are located in the spacer region of pol γA, they occur in an evolutionally conserved domain as depicted in Figure 7. The p.W748 is highly conserved, but p.R722 is not so well conserved even amongst mammals. The PredictProtein and Jpred3 programs do not predict major changes in the secondary structure (data not shown). The tertiary structure on the basis of crystal diffractometric data for the A-type DNA polymerases is known only for *Escherichia coli *POL1 Klenow fragment and bacteriophage T7. Alignment of human pol γA with *E. coli *Klenow fragment of pol 1 identifies a loosely homologous 701-724 region of pol γA. The corresponding region of pol 1 around p.R631 is situated in a crevice being in close interaction of DNA strand in the co-crystallized 1kln.pdb. This region was used as template for homology modeling of pol γA. This puts the p.R722H mutation in a helical region and the p.W748S in a more flexible coil region without major effect on the secondary or tertiary structure (Figure 8).

## Discussion

Heterozygous *POLG1 *p.R722H mutations have previously been reported in two patients with Parkinson's disease and in one control, with no difference in mutation frequencies between these groups [14]. In our study, patient A1 with the homozygous *POLG1 *p.R722H mutation presented with dementia and sensorineural hearing impairment together with progressive external ophthalmoplegia, diabetes mellitus, dysphagia and neuropathic pain in legs. Long PCR and Southern blotting revealed multiple mtDNA deletions (Figure 3 and 4), which have also been reported to be caused by several other *POLG1 *mutations [7,12,13]. Long PCR of the muscle DNA of healthy control samples showed also accumulation of mtDNA deletions, but in obviously minor extent. These deletions are likely due to the aging process and the pattern of deletions is different from the deletions of the patient A1. Long PCR did not show any intact mtDNA in control samples as this method favors the amplification of shorter DNA fragments. On the other hand, Southern blot analysis showed the intact mtDNA, but did not detect deletions in the muscle DNA of control samples.

This combination of multiple mtDNA deletions, multiple neurological and endocrinological symptoms and histochemical findings, including ragged red fibers and COX-negative muscle fibers, suggests a mitochondrial origin for the disease. The clinical features also resemble those reported to be typical of *POLG1 *mutations [3,5,6]. It has been shown in a large cohort of *POLG1 *patients that ptosis and PEO usually presented in teenage years or adult life and additional features included a limb myopathy, ataxia, a peripheral neuropathy or dysphagia, diabetes, deafness and dementia [5]. Our patient A1 manifested with all these typical features associated with *POLG1 *mutations. Two sisters of patient A1 also had the homozygous p.R722H mutation and both presented with dementia and sensorineural hearing impairment. Thus, dementia and sensorineural hearing impairment segregated consistently with the mutation.

We also described the compound *POLG1 *p.R722H + p.W748S mutations in two siblings with mental retardation. The spectrum of clinical findings caused by the p.W748S mutation is wide, and it has been found as a heterozygous mutation compounded with other heterozygous *POLG1 *mutations [5,13,18,29]. Mitochondrial recessive ataxia syndrome (MIRAS) is typically found in patients with homozygous or compound heterozygous p.W748S *POLG1 *mutations who present with adult or juvenile-onset ataxia combined with dysarthria, sensory neuropathy, late cognitive impairment, oculomotor defects, myoclonus, tremor, psychiatric symptoms and seizures [18,29]. The clinical features of patient B1 and her sister (patient B2) are consistent with the patients harboring compound linker region mutations who have epilepsy, mental retardation and psychiatric symptoms as their presenting features [13,18]. A similar phenotype has also been associated with another heterozygous linker area mutation p.G517V with multiple endocrinopathy, basal lacunar infarcts, seizures, headache and psychiatric disorders as presenting features [30]. Recent observations have suggested a possible dominant effect in p.W748S heterozygotes, since more severe phenotypes and poorer survival have been observed in patients with compound heterozygosity for *POLG1 *p.W748S compared with patients who are homozygous for this mutation [18].

Both *POLG1 *mutations, p.R722H and p.W748S, are situated in the linker region which mediates interaction between the polymerase and exonuclease domains, so that mutations might lead to a significant reduction in the enzyme activity *in vitro *[31]. *In vitro *studies have also suggested that the p.A467T mutation leads to conformational changes in the catalytic subunit and disrupts binding of the accessory subunit [32]. The p.W748S mutation alone has been shown to cause a catalytic defect involving poor DNA synthesis and primer extension [33], which suggests that the compound p.R722H + p.W748S mutation in *POLG1 *may lead to dysfunction of the polymerase by alteration of the linker region according to a similar pattern. Although the 3-D structure of subunit-A of the mitochondrial DNA polymerase from higher eukaryotes is not known, the crystal structure of the Klenow fragment (residues 324-928) of *E. coli *DNA polymerase I has been obtained, even complexed with duplex DNA and at 3.2 Å resolution [34]. Beese et al. [34] propose that DNA enters the polymerase site from the 3'-exonuclease domain and bends into a cleft. There are a few arginine residues in this cleft domain, and p.R631 in a region which gives a loose match with the human sequence, and its positively charged guanidinium group is in close contact with the phosphodiester backbone of one of the DNA strands [34]. Site-directed mutagenesis of *E. coli *Klenow fragment has shown that a p.R631A mutation leads to a slight decrease in fidelity of the polymerase reaction although the effect was not statistically significant [35]. Although the p.R722 of the eukaryotes is not highly conserved, the presence of arginine in this region may be important. The homologous p.R631 in *E. coli *is conserved in the Pol I family and located in a domain, which experiences conformational change upon DNA binding. In higher eukaryotes the corresponding "spacer" region shows a high degree of evolutional conservation, suggesting that this region has functional significance. Site-directed mutagenesis of Drosophila pol γA has indicated that the linker domain is involved in DNA binding [36]. Loss of the positive charge of arginine in this region may affect template binding and by this means alter its activity or fidelity.

The carrier frequency of p.R722H was 1:135 being as high as that reported for two other *POLG1 *mutations in the Nordic countries. The carrier frequency of p.W748S is 1:125 in Finland [29] and 1:100 in Norway [13,37], while that of p.A467T is 1:200 in Sweden and in Norway.

## Conclusions

We found a pathogenic mutation p.R722H in *POLG1 *in three siblings, who were homozygous for the mutation and presented with a clinical phenotype suggesting a mitochondrial disease. Interestingly, in another family two children were compound heterozygotes with respect to *POLG1 *p.R722H and p.W748S mutations. The children had mental retardation, ptosis and epilepsy thus resembling the clinical phenotype that has been described in subjects with compound heterozygosity for p.W748S and another *POLG1 *mutation.

Homology modeling and analysis of the *E. coli *pol 1 suggest that p.R722 may reside near enough the DNA-binding site to influence the replication fidelity. Although the conservation of p.R722 is not high, it resides in a highly conserved region. The sequence stretch around p.R722 has some homology with a region in pol 1 of *E. coli *having several amino acid residues with charged side chains probably involved in DNA binding.

We suggest that *POLG1 *p.R722H mutation causes a late-onset neurological phenotype as a homozygous state, whereas the onset of the disease can be earlier in patients with compound heterozygosity for *POLG1 *p.R722H and other pathogenic *POLG1 *mutations. Due to the high carrier frequency of *POLG1 *p.W748S and p.R722H mutations further clinical evaluation of carriers and *in vitro *functional studies of these mutationswill be worthwhile.

## Competing interests

The authors declare that they have no competing interests.

## Authors' contributions

TK participated the molecular genetic studies and drafted the manuscript. RH and SF participated the molecular genetic studies. IH carried out sequence analysis and molecular modeling. MK and LP carried out the clinical evaluation of the patients and wrote the patient cases. HT carried out the histological analysis. HR helped to draft the manuscript. KM and JU conceived of the study, participated in its design and coordination and helped to draft the manuscript. All authors read and approved the final manuscript.

## Pre-publication history

The pre-publication history for this paper can be accessed here:

http://www.biomedcentral.com/1471-2377/10/29/prepub
